# Illuminating the Brain With X-Rays: Contributions and Future Perspectives of High-Resolution Microtomography to Neuroscience

**DOI:** 10.3389/fnins.2021.627994

**Published:** 2021-03-17

**Authors:** Paulla Vieira Rodrigues, Katiane Tostes, Beatriz Pelegrini Bosque, João Vitor Pereira de Godoy, Dionisio Pedro Amorim Neto, Carlos Sato Baraldi Dias, Matheus de Castro Fonseca

**Affiliations:** ^1^Brazilian Biosciences National Laboratory (LNBio), Brazilian Center for Research in Energy and Materials (CNPEM), Campinas, Brazil; ^2^Department of Structural and Functional Biology, State University of Campinas, Campinas, Brazil; ^3^Brazilian Synchrotron Light National Laboratory (LNLS), Brazilian Center for Research in Energy and Materials (CNPEM), Campinas, Brazil

**Keywords:** x-ray microtomography, neurobiology, cell tracing, brain architecture, imaging techniques

## Abstract

The assessment of three-dimensional (3D) brain cytoarchitecture at a cellular resolution remains a great challenge in the field of neuroscience and constant development of imaging techniques has become crucial, particularly when it comes to offering direct and clear obtention of data from macro to nano scales. Magnetic resonance imaging (MRI) and electron or optical microscopy, although valuable, still face some issues such as the lack of contrast and extensive sample preparation protocols. In this context, x-ray microtomography (μCT) has become a promising non-destructive tool for imaging a broad range of samples, from dense materials to soft biological specimens. It is a new supplemental method to be explored for deciphering the cytoarchitecture and connectivity of the brain. This review aims to bring together published works using x-ray μCT in neurobiology in order to discuss the achievements made so far and the future of this technique for neuroscience.

## Introduction

Back in the 1970s, the x-ray tomography technique (also known as “computerized axial tomography,” “transaxial tomography,” and “reconstruction from sections”) was first described as a method used in medical radiography for obtaining a slice through the body of the x-ray absorption with a resolution ranging from 1 to about 2 mm (Hounsfield, 1973; Swindell and Barrett, 1977). This achievement was recognized in the Nobel prize in Physiology or Medicine 1979 (Di Chiro and Brooks, 1979), and since then tomography has been widely used in medical radiology, biological, and material sciences research in order to produce non-invasive, diagnostic, and cross-sectional images of a particular structure within a sample. This technique was further expanded in 1987, when Flannery et al. (1987) developed a so-called “new form of microscopy – microtomography,” based on high-resolution x-ray tomography, producing the first three-dimensional (3D) images of the internal structure of small samples with a micrometer resolution. Microtomography (μCT) can be used to non-destructively create 3D images of internal sections of a sample with a resolution limit comparable to that of a light microscope, allowing not only 3D visualization but also virtual slicing for the obtention of detailed two-dimensional images (2D). Although its roots in computerized axial tomography (CAT or CT) scans have been used for medical imaging for over 40 years, applications of x-ray μCT have been reported for a wide variety of objects (Toda et al., 2008; Chen et al., 2009; Fusseis et al., 2009; Zhu et al., 2011; Tsuchiyama et al., 2013).

In biology, computerized x-ray μCT has provided 3D images of the architecture of biological samples from many species of organisms including mice (Johnson et al., 2006; Fonseca et al., 2018), humans (Bonse et al., 1994; Salome et al., 1999; Mizutani et al., 2010), and insects (Metscher, 2009; van de Kamp et al., 2011; Smith et al., 2016). It has become a common method in studies associated with osteo and dental microstructures (Neues and Epple, 2008; Zou et al., 2011; Davis et al., 2018). Recently, microtomographic studies of soft tissues, which account for a major proportion of biological tissues, have shed light on the structural mechanism of biological functions (Happel et al., 2010; Walker et al., 2014).

However, when it comes to the usage of x-ray μCT for neuroscience, this method remains to be explored. So far, the primary method for the visualization of high-resolution 3D structures of neuronal tissues is confocal light microscopy. Several transgenic strategies for visualizing neuronal network by genetically labeling neurons with fluorescence dyes have been reported (Sakaguchi et al., 2018; Koveal et al., 2020). Nonetheless, transgenic methods cannot be applied to human samples. Although automated serial sectioning along with light and electron microscopic analyses have been proposed as a method for imaging neuronal circuits (Denk and Horstmann, 2004; Micheva and Smith, 2007; Knott et al., 2008; Tapia et al., 2012; Motta et al., 2019), hundreds to thousands of sections must be mechanically prepared to reconstruct a 3D structure of a functionally relevant volume of biological tissue, requiring extensive sample preparation protocol.

One of the first usages of x-ray μCT in the neurobiology field was reported back in 1999 when researchers showed the first 3D images of a rat peripheral nerve using synchrotron-based phase-contrast x-ray μCT (Beckmann et al., 1999). However, cellular resolution using this technique was first obtained in 2009, by Mizutani et al. (2010), revealing the first 3D cellular organization of the neuronal circuits of the human cerebral cortex in a biopsy specimen. Since then, some groups have explored the benefits of this technique to unveil the architecture and organization of neuronal tissues.

Therefore, this review aims to bring together published works using x-ray μCT in neurobiology in order to discuss the achievements made so far and focus on the future of this technique as a promising tool to provide more information about the structural and functional organization of the brain in this current connectomics era.

## Three-Dimensional Imaging Techniques in Neurobiology

The systematic microscopic study of neurons as individualized entities has its beginning in the 19th century with the development of the Golgi staining protocol based on silver impregnation (Golgi, 1873). Before that, early microscopists had studied peripheral nerves and spinal tracts in search for hollow tubes that would hypothetically conduct a fluid abundant with signals from the skin to the brain, and from the brain and spinal cord to muscles (Glickstein, 2006). By the year 1887, using the Golgi staining method, Ramón y Cajal (1911) changed the way neurons were seen (Glickstein, 2006) with his observations and descriptions:

“Against a clear background stood black threadlets, some slender and smooth, some thick and thorny in a pattern punctuated by small dense spots. All was sharp as a sketch with Chinese ink on transparent Japanese paper.”

From this moment on, neuroscientists from all over the world opened the gate of modern neuroscience and improved not only neuroscience itself but also the development of new 2D and 3D imaging techniques in order to explore neural circuits and microscopic anatomy. Imaging has become an important and elusive tool for basic and clinical research due to offering direct and clear obtention of data regarding brain architecture and biochemical activities from macro to nano scales (Ganesana et al., 2017). Over the last few decades, optical methods such as confocal (Erturk et al., 2014) and two-photon microscopy (Oheim et al., 2001; Nimmerjahn, 2012), associated with the use of fluorescent dyes and transgenic fluorescent animals, allowed for not only the morphological analysis of neuronal cells and tissues, but also revealed important biochemical and physiological characteristics of these structures. In addition, *in vivo* imaging has been extensively developed, for example, with two-photon excitation microscopy in the open-skull mouse (Grutzendler et al., 2011; Nimmerjahn, 2012). However, brain tissue is a strong light-scattering medium, which makes it difficult to focus the excitation light on a small target point and detect the emitted signal. Based on this, several optical methods, including BABB (Dodt et al., 2007), SeeDB (Ke and Imai, 2014), Clear (T) or Clear (T2) (Kuwajima et al., 2013), and 3DISCO (Erturk et al., 2012, 2014; Erturk and Bradke, 2013) were developed for 3D imaging.

Nevertheless, axons and dendrites of neurons extend in many directions, some interneurons crossing several millimeters through a huge volume of brain mass, what leads to incomplete tracing and difficulties in the reconstruction of 3D morphology of isolated cells in the whole brain parenchyma. To overcome this, methods for resolving the 3D microstructure of large brain regions are typically based on thin serial slicing and staining of brain sections, followed by imaging numerous individual slices with light or electrons. Although valuable, these procedures still face some issues such as lack of contrast, extensive sample preparation protocols, and the destructive nature of serial sectioning.

As another widely used imaging approach in several fields of science, from bench to the bedside, magnetic resonance imaging (MRI) is also part of the group of methods used for the study of the nervous system, in its three dimensions. Today, MRI can provide numerous contrast modes for various analyses starting at the anatomical contrast between white and gray substances in the central nervous system to tractography or diffusion tensor imaging (Vedantam et al., 2013) to visualize nerve tracts within the brain. The applicability of this technique directly relates to obtaining 2D and 3D structural or functional scanned images of the tissue (Bernard et al., 1988, 2016; Kuehne et al., 2004). However, this approach is an expensive technique, in which image processing is complex and requires time and qualified personnel. In addition, although the imaging of entire structures such as the human cerebellum (Topperwien et al., 2018) could be obtained *in vivo*, the spatial resolution offered by MRI is described as unsatisfactory to study neuronal structures on a cellular/subcellular scale (Schulz et al., 2010; Schulze et al., 2010).

**FIGURE 1:**
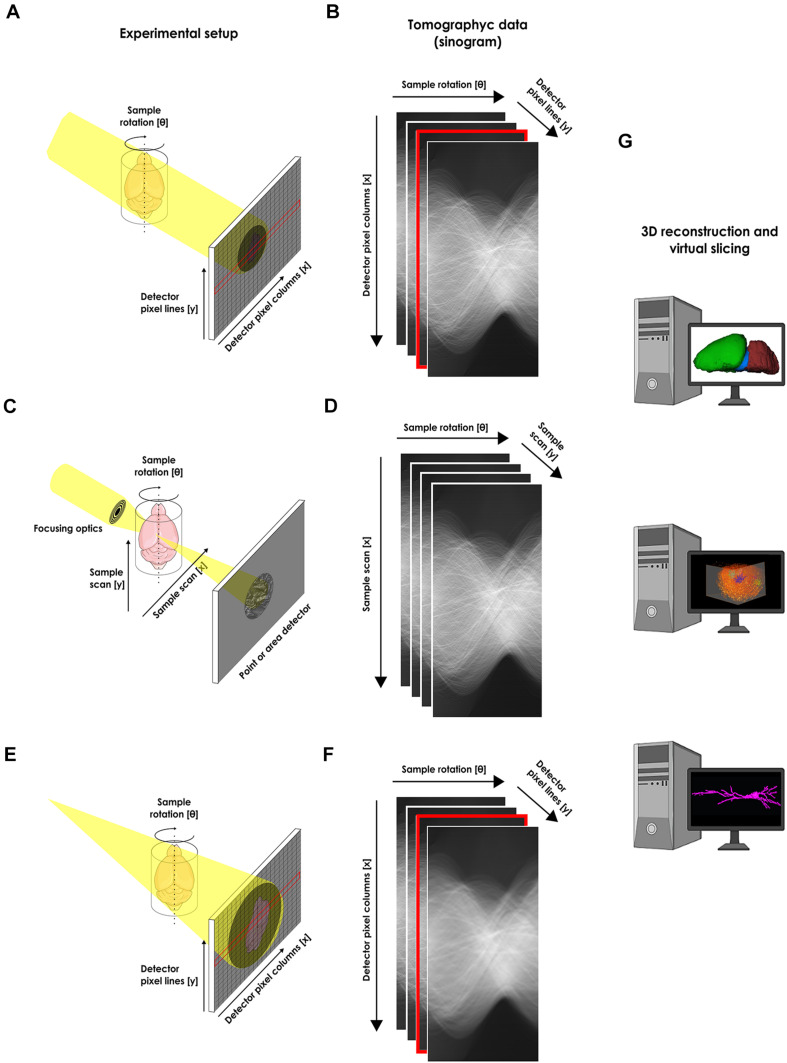
Experimental setups for the structural x-ray microtomographic imaging of the nervous system. **(A)** Schematic view of the experimental setup for synchrotron-based x-ray single-frame μCT. The x-ray from an x-ray source illuminates the whole sample and an x-ray area detector like a CCD (as portrayed), or an x-ray objective-based system (not shown) collects the transmitted projection. **(B)** Recorded single-frame projections organized as a sinogram. **(C)** Schematic view of the experimental setup for scanning synchrotron-based x-ray μCT. The x-ray from an x-ray source is focused on a single point of the sample and a detector collects the signal from a single pixel. The beam scans the whole sample in order to compose an image. **(D)** Recorded scanning projections organized as a sinogram. **(E)** Schematic view of the experimental setup for phase-contrast synchrotron-based x-ray single-frame μCT**. (F)** Recorded single-frame projections organized as a sinogram. In this case, no contrast agent is used during sample preparation and a tomogram is acquired based only on natural contrast of the tissue. An area detector is used for recording the phase shift due to abrupt change in material found on the sample edges. **(G)** After tomographic reconstruction, the obtained tridimensional data can be virtually segmented or sliced for analysis. Cartoons used were obtained from public domain libraries available under the Creative Commons CC0 License (https://creativecommons.org/publicdomain/zero/1.0/).

Therefore, new 3D imaging procedures are still needed to assess increasingly larger brain volumes at the single cell level which makes the use of x-rays an interesting new approach. X-rays can be used to image thick samples, providing a rapid approach for producing large 3D brain maps without sectioning (Figure 1). Hence, to reconstruct the 3D *in situ* morphology of neurons, x-ray μCT became a promising tool.

## High-Resolution Microtomography for the Study of the Nervous System

**FIGURE 2:**
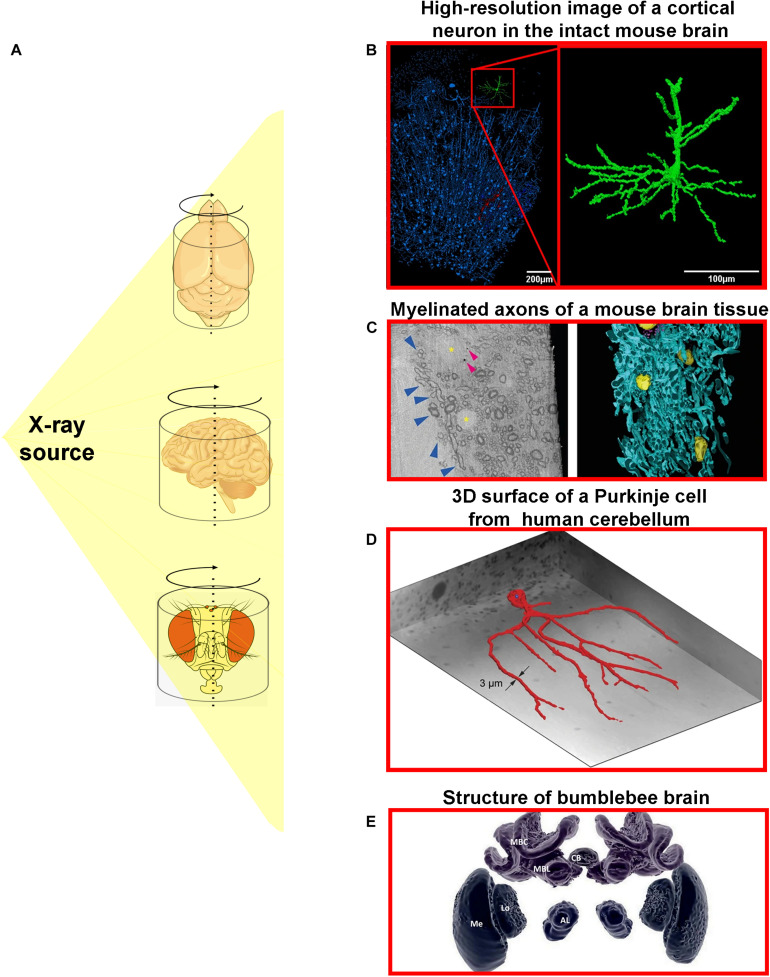
Different x-ray microtomography techniques provide detailed three-dimensional information about nervous tissue architecture in high-resolution. **(A)** The x-ray from an x-ray source can be used to illuminate different samples of nervous tissue from different organisms. **(B)** High-resolution image of a highlighted cortical neuron (green) from the intact mouse brain (Fonseca et al., 2018). **(C)** Cryo x-ray ptychography and 3D color rendering of mouse brain tissue. **(Left panel)** represents a single orthoslice from a reconstructed 3D tomogram. Blue arrowheads show variations of myelin sheath thicknesses of myelinated axons. Yellow asterisks mark multiple cell nuclei and small and roughly spherical structures are pointed by arrowheads. **(Right panel)** portrays the semi-automated color segmentation of the reconstructed tomogram shown in the left panel, based on the contrast differences within the sample. Yellow = nuclei, blue = myelinated axons (Shahmoradian et al., 2017). **(D)** Representation of the 3D surface of a Purkinje cell in the human cerebellum including its dendritic tree (Hieber et al., 2016). **(E)** 3D image of individually segmented brain structures of the bumblebee (*Bombus terrestris*): central body (CB), and one of the pair of lobulas (Lo), medullas (Me), antennal lobes (AL), mushroom body calyces (MBC), and mushroom body lobes (MBL). This figure was adapted from Hieber et al. (2016); Smith et al. (2016), Shahmoradian et al. (2017); and Fonseca et al. (2018) under the Creative Commons License 4.0 (CC-BY) (http://creativecommons.org/licenses/by/4.0/). Cartoons used were obtained from public domain libraries available under the Creative Commons CC0 License (https://creativecommons.org/publicdomain/zero/1.0/).

As previously mentioned, x-ray μCT has been broadly applied for a great number of biological structures especially hard tissue samples such as bones and teeth (Neues and Epple, 2008; Schulze et al., 2010; Zou et al., 2011; Davis et al., 2018). Although still not deeply explored, microtomographic analyses of soft tissues, including nervous tissue, can reveal the functional mechanisms of 3D cellular and subcellular structures based on its spatial organization and distribution (Figure 2). A great example of the importance of spatial organization for organ functioning is well represented by neuronal networks. Understanding how neurons are connected and distributed, from macro- to microscale, is fundamental to unveil brain architecture, functioning, and dynamics.

**FIGURE 3:**
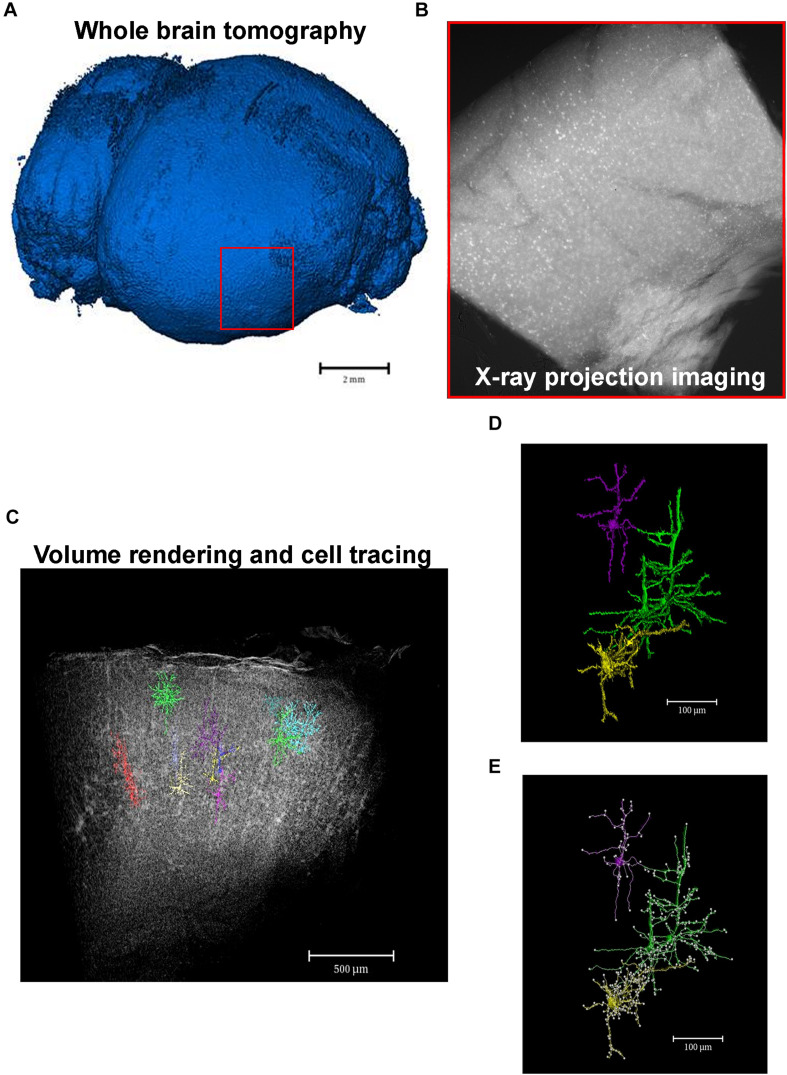
From macro- to microscale imaging in the same set of image acquisition. X-ray μCT allows for the acquisition of images from whole intact organs which can then be virtually sliced and analyzed in a microscale. **(A)** Whole tomography of a paraffin-embedded mouse brain. Red square shows the region of interest to be analyzed in a high-resolution microscale. **(B)** X-ray absorption projection of Golgi-Cox-labeled brain region selected in panel **(A)**. Bright spots represent successfully mercury-impregnated neurons. **(C)** Volume rendering of the whole region selected in panel **(A)** showing some segmented neurons within the structure. **(D)** Cellular segmented neurons of the structure represented in panel **(C)**. Colors highlight a single neuron or a cluster of cells that could not be separated due to close proximity. **(E)** Example of analyzes of neuronal branching patterns. Each white dot represents a cell branch.

X-ray μCT imaging does not require that tissues undergo serial-sectioning or any type of physical processing, i.e., the sample remains completely undamaged, which guarantees that the neuronal circuit is intact and leads to the possibility of building 3D models that have a higher level of detail and resolution (Figure 3).

X-ray imaging based on pure absorption dates back to the discovery of x-ray itself (Zhang and Fuller, 1998). However, a key challenge for using μCT methods for brain imaging is its transparency to x-rays (Masis et al., 2018), thus rendering limited contrast between the different structures within the tissue. Nonetheless, some approaches have already been used to overcome this issue: x-ray fluorescence for elemental imaging and phase contrast and high-z element absorption.

### Scanning X-Ray Fluorescence Microtomography

Scanning X-ray fluorescence (SXF) μCT works in a similar way to a confocal optical microscope as it uses a focused x-ray beam and a scanning probe that detects the fluorescence of a given chemical compound. The essential difference between x-ray fluorescence-CT and conventional CT is that the measurement is not based on the absorption of the incoming x-ray but on the measurement of the fluorescent x-rays generated by a specific chemical element of the sample once illuminated, with the ultimate purpose of reconstructing 3D compositional variations (Figure 1).

Therefore, the measured x-ray fluorescence corresponds to a unique “fingerprint” of a chemical element that provides multiple contrast channels, each related to a different element. It is reported that x-ray fluorescence of a probe element down to a concentration of 1 ppm can be visualized in 3D (Lanzirotti et al., 2009). For intracellular imaging of probe elements (especially transition metals) in delicate biological samples such as brain tissue, recent instrumentation advances in intense x-ray beams of synchrotron x-ray facilities have allowed researchers to achieve nanometer spatial resolution with sub-ppm detection limits for a wide range of elements that may be present in the normal or malfunctioning brain (Collingwood and Freddy, 2017).

The rationale for investigating elementary composition in the brain is diverse. The excellent sensitivity and specificity achievable with fluorescence x-ray microscopy (XRM) allow for the investigation of metal toxicity, for example, from environmental exposure to heavy metals (Md Khudzari et al., 2013). Some studies have also used this technique to investigate the normally functioning brain and disease-mediated changes due to the storage and metabolism of biologically essential metal elements in specific intracellular compartments or as widespread accumulation in the brain. For example, Ortega and collaborators have shown that iron accumulates into dopamine neurovesicles and this process is inhibited by decreased dopamine synthesis (Ortega et al., 2007). In addition, using x-ray fluorescence imaging in conjunction with regular optic microscopy approaches, Carboni et al. (2017) have shown a pronounced difference in trace metal composition of Parkinson’s disease brain samples when compared to the control.

### Phase-Contrast Microtomography

Contrary to absorption contrast μCT, phase-contrast methods can provide increased contrast toward soft tissues as has been shown for a huge array of biological samples (Beckmann et al., 1999; Betz et al., 2007; Chen et al., 2009; Connor et al.,2009a,b; Bravin et al., 2013; Takashima et al., 2015; Topperwien et al., 2018, 2019; Massimi et al., 2019; Shi et al., 2019). Phase contrast aims to exploit the fact that, in the x-ray’s regime, matter is approximately 1,000 times more efficient at refracting than absorbing the x-ray wave. Currently, synchrotron-based phase-contrast imaging partially utilizes coherent x-rays together with an area detector for recording the phase shift due to abrupt change on material found on sample edges. This approach is essentially different from scanning techniques as it illuminates the whole sample recording a single frame for each projection (Figure 1), making the acquisition considerably faster. Therefore, phase-contrast x-ray imaging is especially appropriate for high-throughput 3D characterization of microstructures in biological samples with low x-ray absorption without the need of contrast agents (Bravin et al., 2013; Zhang et al., 2015).

In neuroimaging, the potential of x-ray phase-contrast tomography as a large-scale, label-free, 3D imaging technique has been exploited in a number of recent studies. For example, 3D angioarchitecture of the mouse brain at ultrahigh resolution was revealed using synchrotron-radiation-based propagation phase-contrast imaging (Shi et al., 2019). The effects of ischemic stroke induced in a mouse model by reconstructing the 3D density within the brain tissue was also recently shown (Topperwien et al., 2019). The cerebral angioarchitecture was visualized within large brain regions of rats by Zhang and collaborators (Zhang et al., 2015). Tissue alterations in different disease states, for example neuronal loss and vascular alterations in a multiple sclerosis model (Cedola et al., 2017), or plaque formation in Alzheimer’s disease models (Astolfo et al., 2016; Massimi et al., 2019) have also been addressed. This technique was also applied to *ex vivo* whole brain scans in view of brain tissue morphology for both normal and cancerous tissue, before and after x-ray microbeam radiation therapy (Barbone et al., 2018). A study conducted by Saiga et al. (2019) evaluated the brain and spinal cord of mice with autoimmune encephalomyelitis by x-ray μCT using synchrotron radiation, demonstrating that the spinal cord of animals affected by this condition had a pronounced tissue inflammation portrayed by the observed dilated vessels and vacuolization, the latter also being found in the cerebellum (Saiga et al., 2019). The subsequent reconstruction of a 3D model for the vessels and vacuoles network found that the distribution of vacuoles is not uniform and the more severe the state of the disease, the greater the vasodilatation. Finally, a study by Luo et al. (2019) analyzed brain samples from mice subjected to ischemia and described a new methodology that allowed the 3D analysis of the vascular repair in the ischemic lesion, both qualitatively and quantitatively, so that the morphology of the micro vessels could be visualized after the reconstruction of the vascular skeleton (Luo et al., 2019).

In spite of being a label-free method, resolution and contrast were demonstrated to be sufficiently good for single cell identification in larger volumes both for rodents (Fratini et al., 2015; Bukreeva et al., 2017, 2020) and for human tissues (Hieber et al., 2016; Khimchenko et al., 2018; Topperwien et al., 2018).

### X-Ray Absorption Microtomography Using High-z Probes

High z-probes are contrast agents featuring high-atomic-number (high-Z) elements which absorb x-rays effectively. Examples of these probes that have already been reported for visualizing biological microstructures include osmium (Ananda et al., 2006; Johnson et al., 2006; Litzlbauer et al., 2006; Dias et al., 2019), gold (Mizutani et al., 2007, 2008, 2010; Li et al., 2010), silver (Mizutani et al., 2008; Parameswaran et al., 2009), iodine (de Crespigny et al., 2008; Metscher, 2009), platinum (Wang et al., 2017), mercury (Fonseca et al., 2018; Nani et al., 2020), tungsten (Metscher, 2009), and lead (Kamenz and Weidemann, 2009). The advantage of this method is the clear visualization of structures that may not be well delimited only by phase contrast.

**FIGURE 4:**
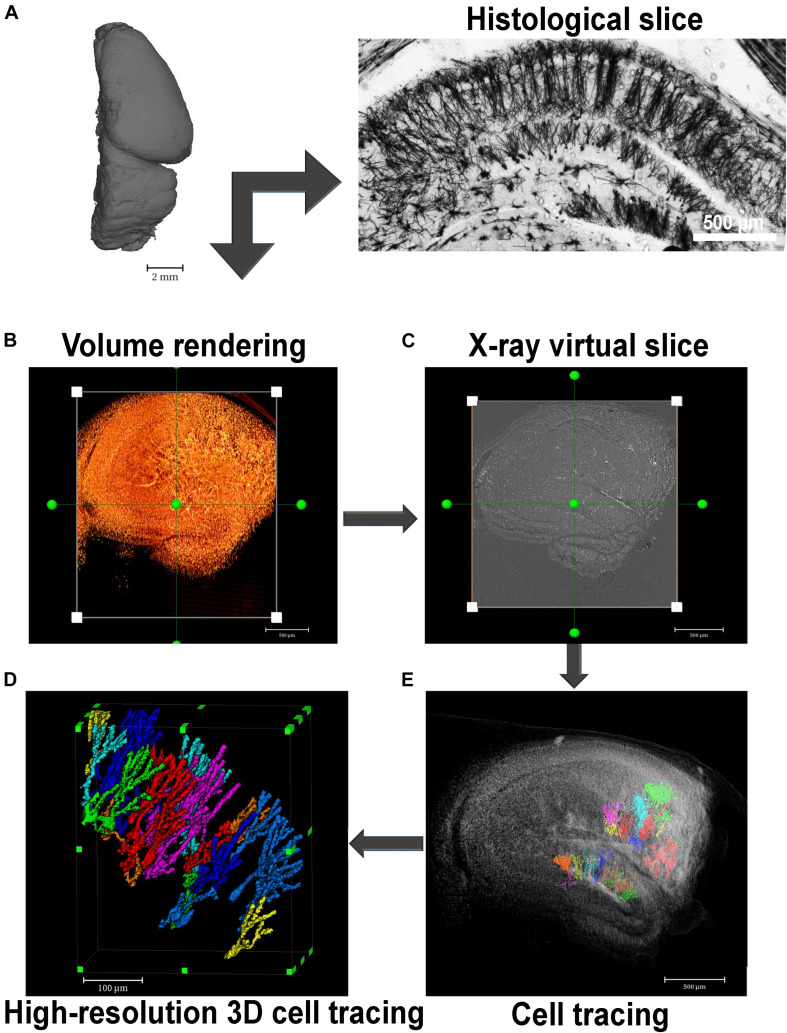
Virtual histology in 3D with x-ray absorption microtomography. **(A)** A paraffin-embedded mouse hemisphere after Golgi-Cox staining and embedded in paraffin can be seen by histology or x-ray microtomography. Far right image shows a histological section of a Golgi-Cox-impregnated mouse hippocampus exhibiting the neurons of the sliced structure evenly and reliably stained. **(B)** This image shows a 3D image rendering of a Golgi-Cox-impregnated mouse hippocampus. **(C)** Virtually reconstructed image of one slice of the rendered image represented in panel **(B)**. **(D)** Volume rendering of some selected slices of the tissue shown in panel **(A)** where some neurons were segmented. **(E)** High-resolution image of segmented neurons located at the dentate gyrus of the mouse hippocampus imaged in panel **(B)**.

So far, recent studies have used high resolution μCT to characterize 3D high-z-labeled structures that make up the central nervous system in various species (Figure 2). Mizutani et al. (2010), using Golgi’s impregnation method, have successfully observed pyramidal neurons, interneurons, and blood vessels within a human cortex brain sample (Mizutani et al., 2010). Using the mouse brain, Depannemaecker et al. (2019) achieved the observation of the blood vessels, brain cortex, lateral ventricle, and cell bodies of neurons (Depannemaecker et al., 2019). Furthermore, the *in situ* morphological characterization of individual neurons in specific brain regions followed by a quantification of cell number from a large volume of the hippocampal tissue, collected from healthy and pilocarpine-treated mice, was recently shown by impregnating the samples with Golgi-Cox solution. This approach was based on impregnating neurons with mercury which enabled a virtual histology, an integrated 3D visualization of the neuronal network, and a 3D quantification of a large portion of tissue (Fonseca et al., 2018; Figures 3, 4). This work was the first to provide visualization of the complete single cell morphology of intact neuronal tissues with x-ray μCT. In addition, using the Golgi–Cox impregnated samples from a rat model with dysfunctional disrupted-in-schizophrenia-1 signaling, the assessment of neuronal cell body number and the spatial organization of brain neurons revealed defective neuronal positioning, characteristic of impaired cell migration, in striatum/nucleus accumbens and the prefrontal cortex compared to wild-type brains (Nani et al., 2020). Dyer et al. (2017), in turn, identified the 3D architecture of neurons and glia cell bodies, vasculature, large segments of apical dendrites, and myelinated axons via staining of brains with heavy metals (Dyer et al., 2017).

Another type of sample widely used in μCT neuroimaging *is Drosophila melanogaster* brains impregnated with heavy metals such as gold and silver. Some studies have resulted in the construction of a 3D model of the neural network skeleton of this organism that could allow for the obtention of valuable information about the functional mechanisms of the brain (Mizutani et al., 2007, 2008). Recently, the obtention of images using a combination of x-ray absorption and phase-contrast effects was reported (Chin et al., 2020). Specifically, for the portion of the neurons labeled by the Golgi–Cox staining in each specimen, absorption contrast dominated. Instead, other features like the *Drosophila* skull are primarily revealed by phase contrast. This difference facilitates the reliable identification of stained neurons and other neuron-related structures.

## Perspectives

### Instrumentation

The contribution to neuroscience development using x-rays, especially based on synchrotron radiation, is deeply related with the development of XRM in its various forms. It is well known that the deep relation between the shape and function of biological structures makes the brain one of the most complex hierarchical structures found in nature with significant structures down to the sub-cellular level. Accordingly, those structures can only be fully understood if a proper 3D imaging technique is available. Within this context, XRM refers to a collection of imaging techniques with enough spatial resolution and high penetration power for 3D imaging.

All these approaches rule out the need for fluorescence dyes and complex sample preparation. Nonetheless, XRM is still in an initial state of development creating the necessary tools focusing not only on instrumentation but also on sample preparation and mounting protocols (Dias et al., 2019).

Different types of XRM such as full-field x-ray microscopy and ptychography can provide improved spatial resolution when compared to super-resolution optical microscopy. Also, advanced contrast modes exploring chemical speciation and nanoscale assembly would provide new 3D mapping of brain tissue organization. From the sample preparation perspective, the development of specific staining procedures would allow functional imaging similar to light microcopy but with increased resolution.

### Spatial Resolution

X-ray microscopy can be divided into two main categories: single frame, where an entire image is collected with a single exposure; and scanning, where each pixel on the image is measured individually (Figure 1). Naturally, the former is considerably faster than the latter and in the case of tomography, when a collection of multiple images is necessary, the acquisition time of a single image rapidly scales and becomes of great importance.

Recent developments in x-ray optics have allowed the production of x-ray objectives, opening the way for a lens arrangement similar to an optical microscope. This technique offers rapid nanoscale x-ray imaging capability, meaning tomographic acquisitions in the order of minutes per sample (Weinhardt et al., 2019) and a spatial resolution as small as 20 nm. So far, most of the advances for this approach were made in the soft x-ray energy range (named water window) for exploring the existent natural contrast between protein and water within this energy range. However, this also limits the x-ray penetration depth to 10 μm making this a suitable tool for single cell image only (Sakdinawat and Attwood, 2010; Hemonnot and Koster, 2017). For tissue samples, high energy x-ray microscopes would allow for the imaging of samples as big as 1,000 μm but with the cost of losing the absorption contrast for biological samples. However, as demonstrated by Holzner et al. (2010), hard x-ray full-field microscopes can be equipped with a Zernike phase plate (ZPP) for phase contrast. Indeed, Yang et al. (2010) and Vartiainen et al. (2014, 2016) have demonstrated that this approach is able to achieve a spatial resolution of 50 nm on biological samples.

However, the hierarchical structure of a brain also presents structures that are smaller than 50 nm. In that sense, the use of high x-ray with shorter wavelengths would also contribute toward increasing resolution. Current developments on XRM still limit hard x-ray full-field imaging to a 30 nm resolution (Weinhardt et al., 2019), especially due to hard x-ray lens quality since higher energy x-ray optics are much more challenging to manufacture. This led to the independent development of lensless XRM techniques known as coherent diffraction imaging (CDI; Lo et al., 2018; Nakasako et al., 2020). These techniques can achieve a substantially better spatial resolution by eliminating the need for a hard x-ray objective, which is promising for the visualization of neuronal structures much smaller than 50 nm. In general, CDI measures the scatted x-ray wave of a sample meaning the sample’s phase information will be encoded in the form of a diffraction pattern. By measuring this pattern on an area detector, the sample can be reconstructed using specialized phase retrieval algorithms. Likewise, the phase retrieval algorithms together with the area detector, replace the need for an objective lens. On the other hand, CDI depends on a highly coherent x-ray source, meaning CDI-based XRM usage is restricted to synchrotron-light facilities.

There are several CDI methods namely plane-wave CDI, Bragg CDI, and ptychography, among others (Lo et al., 2018; Nakasako et al., 2020). Ptychography is a scanning microcopy technique consisting of a series of diffraction patterns from partially overlapping regions that allows for robust image reconstruction with both absorption and phase-contrast imaging (Stockmar et al., 2015). Examples of both 2D imaging (Gallagher-Jones et al., 2017) and 3D ptycho-tomography of cells (Deng et al., 2018) already attest for the feasibility of the technique on achieving resolutions below 20 nm on biological samples. It is worth mentioning a work done by Shahmoradian et al. (2017). In this work, the authors used hard x-ray ptycho-tomography to image a chemically fixed mouse brain tissue in a frozen-hydrated state without heavy metal staining and reached a 3D resolution of ∼120 nm on a volume of 48 μm^3^ during a 23-h imaging acquisition. Such time-consuming measurement is due to the scanning characteristic of the technique. However, with the new 4th generation synchrotron sources already in operation the coherent photon flux necessary for this type of experiment should increase 1,000-fold, meaning 1,000-fold faster experiments.

### Specific Staining

So far, few cell- or tissue-specific contrast agents for μCT are reported in literature (de Bournonville et al., 2019). Therefore, the development of novel tissue-specific contrast agents still needs to be widely explored in this field. An attempt in that direction was beautifully performed with whole kidney eosin-based staining. In this work, the authors presented a cytoplasm-specific staining method tailored for x-ray μCT that enables a routine and efficient 3D volume screening at high resolutions (Busse et al., 2017).

Following this cell-specific contrast agent idea, some studies demonstrated the applicability of 40-nm biocompatible bismuth particle cores encapsulated in poly(lactic-*co*-glycolic acid) (PLGA) (Naha et al., 2014; Swy et al., 2014; Zhu et al., 2014) that enabled efficient targeting of the particles to cancer cells that overexpress folic acid receptors. These labeled cells could then be imaged *in vitro* or *in vivo* in a xenograft tumor model (Zhu et al., 2014). Another reported example is the use of iron nanoparticles for single cell visualization (Kim et al., 2017). Because of the limited spatial resolution and low affinity of the nanoparticles to the cells, the minimum number of detectable cells was 50,000 within muscle samples. In addition, the nanoparticles needed to be administered inside the cells before implantation in the tissue, questioning the potential cytotoxicity of this method as an effective contrast agent.

In neurobiology, the examples are even scarcer. Although several contrast agents are used to label nerve tissues for μCT these reagents stain neurons randomly, which makes it impossible to distinguish among specific neuronal populations based on neurotransmitter content, for example. Recently, one technique showed that 20-nm gold nanoparticles conjugated to antibodies, routinely used in the immunohistochemistry method, to specifically label neuronal nuclei (Depannemaecker et al., 2019). However, resolution was very low, and improvements are necessary to increase image quality and the application of this method. Hence, future developments should focus on designing, synthesizing, and evaluating new high-affinity, neuron-specific contrast agents. Some possibilities may be considered, for example, the development of contrast agents for x-ray that bind specifically to membrane proteins or that are transported to the cytoplasm by specific membrane transporters, which would generate so-called “immunological imaging.” To the best of our knowledge, literature regarding immunological imaging in μCT is, however, premature. This 3D high-resolution immuno-specific-based μCT can be highly valuable since it could provide relevant information about the spatial distribution of specific cell types, proteins, or antigens within 3D heterogeneous tissues. Because of the high potential of these novel protein/antigen-specific contrast agents, we believe that this approach should be deeper explored and will generate groundbreaking findings from their use in neurobiology.

### Imaging Analysis

Although constant refinement and development of imaging acquisition methods are in progress, future directions in the development of μCT for biological research must also focus on developing computational and analytical tools in order to make full use of the power of quantitative 3D images. Computational reconstruction of neural circuits within tissues from big volume data such as those obtained by x-ray μCT require the tracing of cells in their totality, including all their neurites. Most of the automated approaches that have been developed so far for cell tracing still face some considerably high error rates in order to generate reliable circuit diagrams and demand extensive human supervision. Although most of the analytical approaches used for 3D image analysis of big volume data were developed for electron microscopy images, several can also be applied to μCT data, as will be discussed.

A flood-filling network, for example, is a method developed for automated segmentation that uses convolutional neural networks together with a recurrent pathway that allows for the iterative optimization and extension of individual neuronal processes (Januszewski et al., 2018). Another promising method named SegEM was recently presented and consists of a toolset for efficient semi-automated analysis of large-scale fully stained 3D-EM datasets for the reconstruction of neuronal circuits (Berning et al., 2015). By combining skeleton reconstructions of the imaged neurons with automated volume segmentation, this toolset allows for the reconstruction of neuronal circuits at a work hour consumption rate of about 100-fold less than that necessary for manual analysis and about 10-fold less than existing segmentation tools, as stated by the authors.

In addition to imaging analysis, ongoing advances in tomographic reconstruction algorithms (Padole et al., 2015) are allowing high-quality data to be collected with lower doses and in shorter timescales, which is of particular importance to μCT studies of biological soft tissues especially dynamically changing living tissues.

## Concluding Remarks

Although in development for over 50 years, the μCT field is still very young, but with high potential for biomedical research, especially for neuroscience. Until now, x-ray μCT applied to biological samples has revealed *in situ* cellular/subcellular structures and their spatial distribution in a 3D matrix that are fundamental to understanding features of an organ that are relevant to many biological functions. However, the convergence of sciences for the physics of x-rays, x-ray microscopy, biology, and 3D structure analysis in neuroscience still need to be thoroughly explored with the promise to break new ground using this interdisciplinary approach.

To our knowledge, this is the first integrative review that gathered published works focusing on μCT applied to neurobiology. One can see that there is a strong need for the development, optimization, and validation of specific contrast methods when it comes to heterogeneous tissues such as the brain and this will only be fulfilled with an integrative approach. In addition, image analysis demands further improvement to generate complete brain maps with cellular resolution and speed up the connectomics era.

Once accomplished, and with the new upcoming 4th generation of synchrotron-light sources in Europe and South America, imminent revolutionary contributions of μCT to the neurobiology field, from basic, clinical, and translational research, can be expected.

## Author Contributions

MF and CD supervised the study and conceptualized the idea. All authors wrote the manuscript, contributed to the article, and approved the submitted version.

## Conflict of Interest

The authors declare that the research was conducted in the absence of any commercial or financial relationships that could be construed as a potential conflict of interest.
